# Adaptation reveals multi-stage coding of visual duration

**DOI:** 10.1038/s41598-018-37614-3

**Published:** 2019-02-28

**Authors:** James Heron, Corinne Fulcher, Howard Collins, David Whitaker, Neil W. Roach

**Affiliations:** 1 Bradford School of Optometry and Vision Science, University of Bradford, BD7 1DP Bradford, UK; 2 Visual Neuroscience Group, School of Psychology, The University of Nottingham, Nottingham, NG7 2RD UK; 3 School of Optometry & Vision Sciences Maindy Road, Cathays, Cardiff University, Cardiff, CF24 4HQ UK

## Abstract

In conflict with historically dominant models of time perception, recent evidence suggests that the encoding of our environment’s temporal properties may not require a separate class of neurons whose raison d'être is the dedicated processing of temporal information. If true, it follows that temporal processing should be imbued with the known selectivity found within non-temporal neurons. In the current study, we tested this hypothesis for the processing of a poorly understood stimulus parameter: visual event duration. We used sensory adaptation techniques to generate duration aftereffects: bidirectional distortions of perceived duration. Presenting adapting and test durations to the same vs different eyes utilises the visual system’s anatomical progression from monocular, pre-cortical neurons to their binocular, cortical counterparts. Duration aftereffects exhibited robust inter-ocular transfer alongside a small but significant contribution from monocular mechanisms. We then used novel stimuli which provided duration information that was invisible to monocular neurons. These stimuli generated robust duration aftereffects which showed partial selectivity for adapt-test changes in retinal disparity. Our findings reveal distinct duration encoding mechanisms at monocular, depth-selective and depth-invariant stages of the visual hierarchy.

## Introduction

When interacting with our environment we continually monitor event duration. For example, when watching streamed video content, the quality of our viewing experience is greatly degraded by ‘packet loss’ leading to the stream’s ‘stalling’ or ‘freezing’^1^. The extent of this perceptual degradation increases sharply as the stalling duration increases between 0–1000 ms^2^. Duration-dependency of this kind is just one example of perception underpinned by the rapid, accurate encoding of sub-second visual durations. Although this encoding often yields duration estimates that are both noisy^3,4^ and distorted^5,6^, the lack of any duration-specific sensory receptor surfaces arguably makes it surprising that it can be completed at all.

Uncertainty about how the nervous system computes duration has led to an assortment of candidate mechanisms whose proposed neural loci range from the pre-cortical to the association cortices. The most peripheral examples are founded in the properties of pre-cortical neurons with circular, small-diameter spatial receptive fields. These models provide explanations for visual temporal distortions with very narrow (~1°) spatial tuning and no specificity for visual orientation^7–9^. A pre-cortical locus for duration processing is given further credence by studies where temporal perception has been measured in patients whose inter-hemispheric communication is prevented via commissurotomy^10^. These patients can show normal cross-hemispheric duration discrimination performance alongside marked deficits in cross-hemispheric spatial discrimination^11^. This strongly suggests pre-cortical processing of duration which then projects bilaterally to both cortical hemispheres^12^.

Alternatively, several lines of evidence point towards duration encoding being a cortical phenomenon. For example, training-induced improvement of duration discrimination sensitivity transfers fully between locations spanning trained and untrained visual hemispheres^13^. Moreover, some perceptual distortions of perceived duration show broad^14^ or non-existent^15,16^ spatial tuning. Where spatial tuning *has* been reported, its characteristics have variously included dependency on stimulus size^14^ or spatiotopic position^17,18^ (but see^19^). In some cases, orientation specificity has also been reported, consistent with the orientation tuning profiles of striate cortical neurons^20,21^.

Although these findings are suggestive of a cortical basis for duration processing, uncertainty persists about whether they are examples of specificity for duration or other perceptual parameters. For example, the cross-hemisphere transfer of training-induced sensitivity improvements could simply reflect non-specific reductions in high-level decisional noise^22^, rather than improvements in duration encoding accuracy *per se*. Similarly, whilst a lack of orientation specificity could reflect a (pre-cortical) processing stage that precedes orientation encoding it is also consistent with downstream stages where orientations are pooled (e.g.^23,24^) to provide high-level specificity for object categories^25^. Finally, experimental manipulations designed to test the spatial specificity of duration distortions have the unintended consequence of manipulating the locus of spatial attention (e.g., adapting to the temporal properties of a stimulus presented at one location then testing at a different location). Given that spatial attention itself strongly influences perceived duration^26–28^, it likely contributes to the presence or absence of spatial tuning via the attentional modulation of (a) adapting and/or test stimulus’ duration, or (b) gain adaptation amplitude itself.

In the current study, we address the question of whether duration encoding has a localised position within the visual processing hierarchy. To do so, we combine the technique of sensory adaptation with methodology that allows us to utilise the anatomical progression from monocular, binocular, depth-selective and depth-invariant processing. Observers adapted to repeated presentations of relatively long or short sub-second visual durations. In keeping with earlier reports^29,30^ adaptation generated bidirectional duration aftereffects: subsequently viewed test durations were perceptually expanded/contracted in the opposite direction to the adapting stimulus.

Using dichoptic presentation, adapting and test stimuli were isolated within monocular channels. This allowed us to test for duration aftereffect selectivity within processing stages at or above those underpinned by neurons in V1: the major recipient of geniculate input^31^ and the first site of anatomical convergence between the eyes^32^. We found robust interocular transfer: substantial duration aftereffects persisted when adapted or non-adapted eyes viewed a luminance-defined test stimulus. Nevertheless, aftereffects failed to transfer completely between the eyes, revealing a small but significant contribution from monocular channels. We then systematically tested the relationship between duration selectivity and more sophisticated levels of binocular processing. To do so, we deployed a novel stimulus which allowed the presentation of durations defined solely by inter-ocular retinal disparity. Despite being completely invisible to monocular mechanisms, these durations generated robust duration aftereffects. Finally, we demonstrate that disparity-defined duration aftereffects show limited transfer across large adapt-test differences in disparity-defined depth plane. This transfer exposes a downstream contribution from a mechanism which encodes duration after pooling across its disparity-selective afferents. These findings indicate that visual duration processing cannot be explained by sub-cortical mechanisms alone. Instead, it receives input from multiple processing stages, consistent with duration being a generic stimulus property whose encoding is shared across mechanisms located both upstream and downstream from the site of binocular integration.

## Results

**Figure 1:**
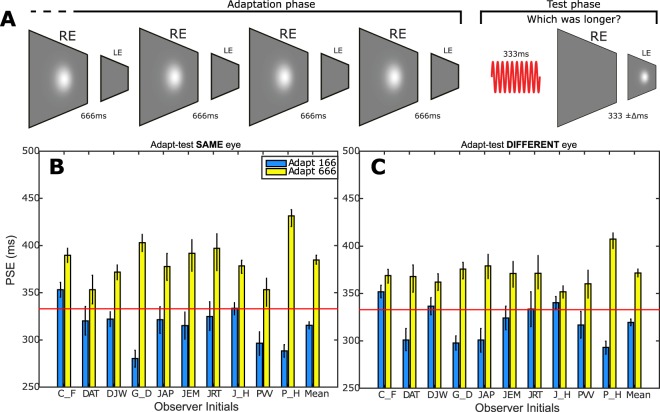
Monocular vs binocular contributions to duration processing. (**A**) Schematic showing the final four ‘top-up’ (fixed duration) adapting stimuli. The subsequent test phase comprised a fixed duration auditory tone (red sinusoid) followed by a variable duration visual test stimulus presented to the same or opposite eye. In the above example of a ‘different eye’ trial, relatively long duration adapting stimuli are presented to the right eye only followed by the presentation of moderate duration test stimuli to the left eye only (see Methods for details). (**B**,**C**) Individual and group mean Point of Subjective Equality (PSE) values representing the test durations that were perceptually equivalent to the auditory reference stimulus after adaptation to relatively long (666 ms - yellow bars) or short (166 ms - blue bars) visual durations. Longer PSE values reflect perceptual compression of test stimulus duration. Red horizontal lines denote veridical duration perception. Data are shown for conditions where adapting and test stimuli were presented to the same (**B**) or different (**C**) eyes. Throughout, error bars represent bootstrapped 95% CIs.

We began by assessing the extent to which the mechanisms contributing to duration aftereffects exhibit elementary binocularity. During the adaptation phase (see Fig. 1A and Methods for details), observers viewed repeated presentations of isotropic, luminance-defined Gaussian blobs. All stimuli were presented via a two-mirror stereoscope which allowed adapting and test stimuli to be presented to the same or different eyes. The duration of these visual stimuli was fixed at relatively long (666 ms) or short (166 ms) durations which were held constant within an experimental session. Following adaptation, the test phase comprised a duration discrimination judgment between a fixed duration 333 ms reference stimulus (auditory tone) and test stimulus (Gaussian blob) whose duration varied around 333 ms.

When these judgments were plotted as a function of test duration, the resultant psychometric functions allowed extraction of the Point of Subjective Equality (PSE): the physical test duration perceptually equivalent to reference stimulus (Fig. 1B and C). Longer/shorter PSE values reflect adaptation-induced perceptual contraction/expansion of test duration, respectively. Differences between PSE values corresponding to the ‘adapt 166 ms’ and ‘adapt 666 ms’ conditions reflect the magnitude and direction of any duration aftereffect. When the adapting and test stimuli were both presented to the same eye, adaptation to relatively long durations induced perceptual contraction of the moderate duration test stimulus. This can be seen in Fig. 1B (yellow bars): post-adaptation, longer duration test stimuli (and thus longer PSE values) were required to maintain perceptual equivalence with the 333 ms reference stimulus. The reverse is evident following adaptation to relatively short durations (blue bars). This pattern of perceptual distortion represents a repulsion-type after effect: adaptation distorts perceived duration in the opposite direction to the adapting stimulus. Consistent with earlier reports^29,33,34^, this effect is asymmetrical around the 333 ms reference stimulus value (Fig. 1B and C, horizontal red line), reflecting the fact that auditory durations are typically perceived as being longer than their (physically identical) visual counterparts, irrespective of adaptation. Importantly, a similar pattern of duration aftereffects can also be observed when adapting and testing durations are presented to opposite eyes (Fig. 1C).

**Figure 2:**
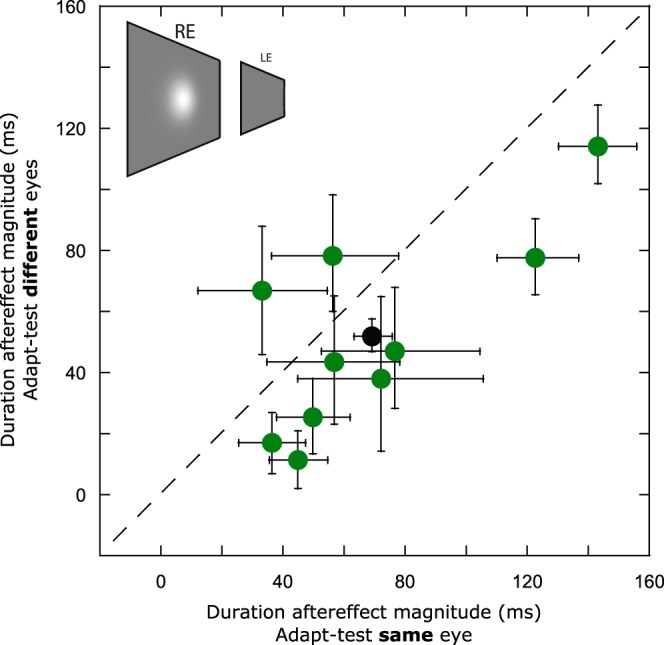
Scatter plot showing data for individual (green circles) and group mean (black circle) duration aftereffect magnitudes representing the arithmetic difference between PSEs extracted from 666 ms and 166 ms adapting durations in the ‘same eye’ (Fig. 1B) and ‘different eye’ (Fig. 1C) conditions. The dashed diagonal line represents complete interocular transfer of duration aftereffects from adapted to non-adapted eye. Error bars represent bootstrapped 95% CIs.

The arithmetic difference between PSEs for each adapting duration provides a measure of duration aftereffect magnitude. Plotting these values for each observer’s ‘same’ and ‘different’ eye conditions (Fig. 2 - green circles) allows visualisation of any systematic trends towards greater duration aftereffect magnitude values in either condition. Averaged across observers, duration aftereffect magnitude values were 69 ms in the “same eye” condition and 52 ms for the “different eye” condition (Fig. 2, black data point). Permutation testing showed that both values were significantly different from zero (p < 0.001) and that the difference between them (Fig. 2 - black circle’s departure from the diagonal) was also highly significant (p < 0.001). These data therefore represent duration aftereffects mediated by mechanisms that are predominantly binocular, alongside a small but significant monocular component.

Despite some evidence for modest inhibitory interactions between eye inputs at the level of the LGN^35,36^, the first examples of excitatory convergence upon binocular neurons are found in the superficial and deep layers of V1^37^. The aftereffect’s small monocular component could therefore reflect pre-cortical adaptation^38^ or adaptation within monocular V1 (i.e. layer 4 C^39^) neurons. The much larger binocular component could be mediated within cortical neurons showing rudimentary binocularity (i.e. responsivity to binocular stimulation but limited integration of monocular inputs) or more sophisticated forms of binocular processing such as the extraction of retinal disparity. In primate V1 for example, approximately 50% of binocularly responsive neurons show selectivity for retinal disparity information that can only be extracted by comparing the spatial properties of their monocular inputs^40,41^. To test whether the binocularity shown in Fig. 2 allows the extraction of duration information that is invisible to monocular channels we measured the effects of adapting to durations defined solely by the presence or absence of crossed retinal disparity.

**Figure 3:**
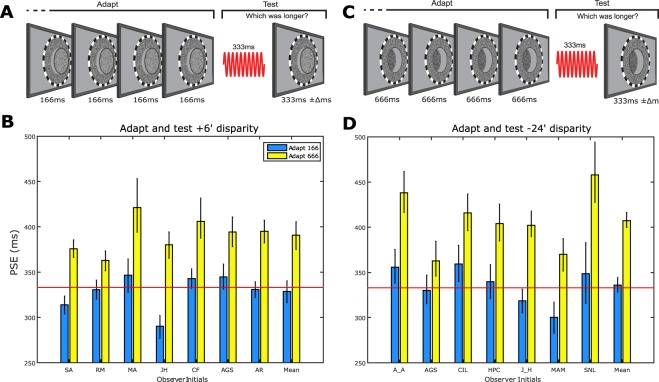
Adaptation to durations defined by retinal disparity. (**A**) Schematic representing observer’s perception of disc-shaped (fixed duration) adapting and (variable duration) test stimuli whose durations were defined by periods of +6′ (crossed) retinal disparity and zero disparity inter-stimulus intervals (see Methods for details). In this example, relatively short adapting durations precede the presentation of modulate duration test stimuli. (**B**) Individual and group mean PSE values derived from conditions where the 6′ disparity-defined, variable duration test stimuli were presented following adaptation to relatively long (666 ms - yellow bars) or short (166 ms - blue bars) 6′ disparity-defined adapting stimuli. (**C**) As per (**A**), except that adapting and test durations were defined by periods of −24′ (uncrossed) retinal disparity. In this example, relatively long adapting durations precede the presentation of modulate duration test stimuli. (**D**) As per (**B**), except that PSEs were derived from conditions where adapting and test stimuli were both presented with −24′ retinal disparity. Red horizontal lines denote veridical duration perception. Throughout, error bars represent bootstrapped 95% CIs.

By presenting the disparity only during the periods corresponding to adapt/test stimulus durations, we excluded monocular contributions to perceived duration. Observers viewed left and right stimuli simultaneously via a mirrored stereoscope. These images were identical except for a lateral shift within a subset of pixels (see Methods) which defined a disc shaped region of +6′ crossed retinal disparity (Fig. 3A). To prevent monocular artefacts (e.g., positional shift cues), we embedded the disparity within dynamic luminance noise (see Methods) which precluded monocular tracking of pixel positions around the time of the disparity’s introduction/removal (i.e. the disparity-defined duration’s onset/offset). The disparity was presented for intervals matching the required adapting or testing duration and therefore disappeared during the (zero disparity) inter-stimulus intervals. The disparity-defined adapting and test stimuli were identical except for their differences in duration. Adapting and test durations matched those used in the interocular transfer experiment.

The resultant PSE values for each adapting duration are shown in Fig. 3B. Despite the invisibility of adapting and test stimuli to monocular mechanisms, all observers show a robust pattern of duration aftereffects. Specifically, adaptation to relatively long/short disparity-defined durations causes significant (p < 0.001) perceptual contraction/expansion of subsequently view disparity-defined test durations. Taken together with the results of the inter-ocular transfer experiment, this finding suggests that duration encoding must occur within at least two processing stages: one monocular and a second with selectivity for duration, *and* retinal disparity.

In the primate visual system, the proportion of disparity-tuned neurons increases progressively from V1 through V2-V3/V3A^42^, MT^43^ and V4^44^. However, higher-order visual processing in V4^45^, inferotemporal^46^, intraparietal^47,48^ and lateral occipital^49^ cortices shows neuronal selectivity for object shape that is invariant across changes in retinal disparity. In many cases, these object shapes are defined by their three-dimensional structure, suggesting that this structure is constructed via pooling across upstream disparity-selective inputs^50^. It therefore follows that if duration aftereffects fail to transfer across adapt-test changes in stimulus depth plane the locus of the underlying mechanism would be constrained to processing stages between the encoding of, and pooling across, retinal disparity.

**Figure 4:**
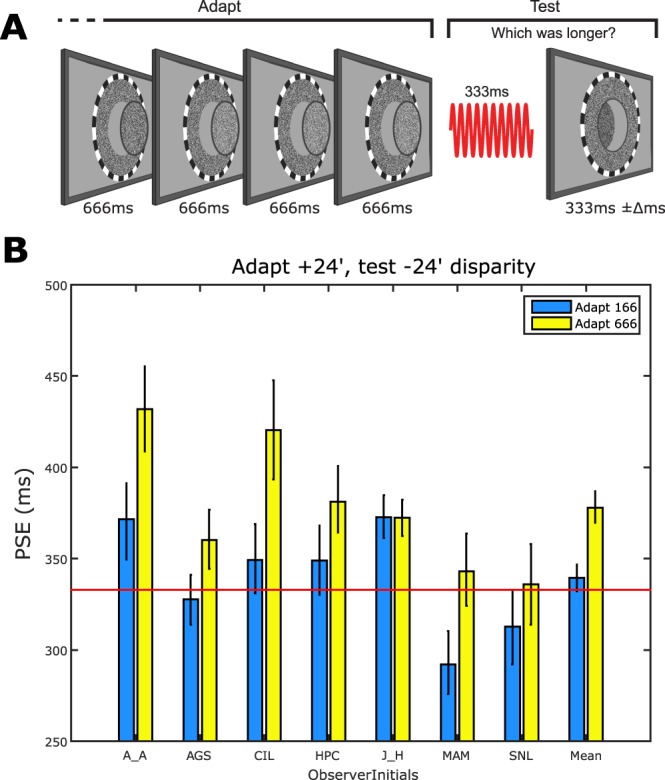
Duration adaptation across adapt-changes in retinal disparity. (**A**) Schematic showing observer’s perception of adapting stimuli defined by +24′ (crossed) retinal disparity and test stimuli defined by −24′ (uncrossed) retinal disparity. (**B**) Individual and group mean PSEs derived from conditions where variable duration −24′ disparity-defined test stimuli were presented following adaptation to relatively long (666 ms - yellow bars) or short (166 ms - blue bars) +24′ stimuli. The red horizontal line denotes veridical duration perception. Throughout, error bars represent bootstrapped 95% CIs.

We tested this hypothesis by measuring duration aftereffects across a 48′ difference in the retinal disparity defining the adapting and test stimuli (Fig. 4A). Test stimuli were always presented with −24′ retinal disparity whereas adapting stimuli were presented with either the same (−24′ (Fig. 3C)) or different (+24′ (Fig. 4A)) retinal disparity (see Methods for details). We selected these values to minimise the possibility that adapting and test stimuli might stimulate common populations of neurons with broad disparity tuning (Parker, 2007). Figure 3D shows PSE values for the ‘same disparity’ conditions. Following adaptation to durations defined by uncrossed retinal disparity, all observers show duration aftereffects comparable with those observed following adaptation to luminance defined (Fig. 1B) and crossed retinal disparity defined (Fig. 3B) durations. This equivalence suggests that duration adaptation may be insensitive to the nature of the disparity defining the adapting and/or test stimulus’ duration. When this possibility is tested by the introduction of a large adapt-test retinal disparity difference (Fig. 4A) significant duration aftereffects persist (Fig. 4B), albeit of a substantially reduced magnitude relative the ‘same disparity’ condition (Fig. 3D). This reduction represents a degree of selectivity for retinal disparity.

**Figure 5:**
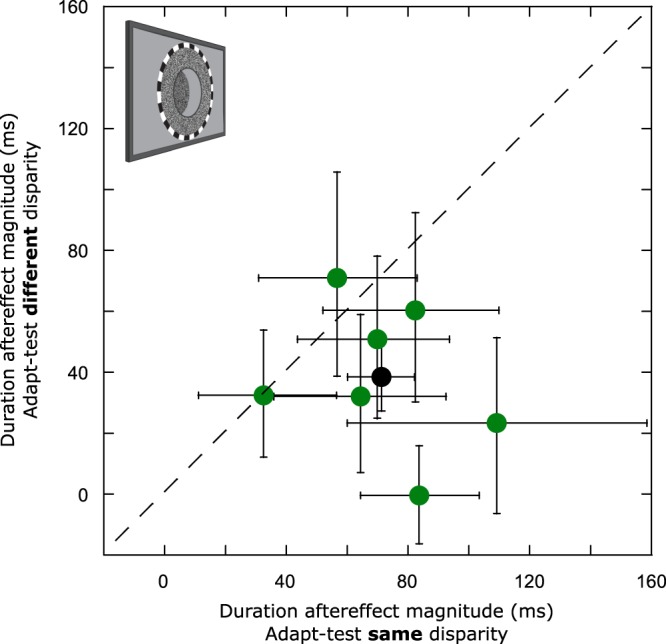
Scatter plot showing data for individual (green circles) and group mean (black circle) duration aftereffect magnitudes representing the arithmetic difference between PSEs extracted from 666 ms and 166 ms adapting durations in the ‘same disparity’ (Fig. 3C and D) and ‘different disparity’ (Fig. 4A and B) conditions. The dashed diagonal line represents complete transfer of duration aftereffects from +24′ (crossed) adapting stimuli to −24′ (uncrossed) test stimuli. Error bars represent bootstrapped 95% CIs.

This selectivity is highlighted when Figs 3D and 4B’s PSE data is expressed as duration aftereffect magnitude (Fig. 5). For example, all observers show significant duration aftereffects when adapting and test stimuli’s durations are defined by the same retinal disparity (horizontal distance between Fig. 5’s green data points and the y-axis) but for 6 out of 7 observers these aftereffects are of reduced magnitude when adapting and test stimuli are defined by different retinal disparities. Averaged across observers, duration aftereffect magnitude values were 71 ms in the “same disparity” condition and 39 ms for the “different disparity” condition (Fig. 5, black data point). Permutation testing showed that both values were significantly different from zero (p < 0.001) and that the difference between them (Fig. 5 - black circle’s departure from the diagonal) was also highly significant (p < 0.001).

## Discussion

We sought to investigate the binocularity of human duration perception. We used sensory adaptation techniques to generate duration aftereffects. We tested the specificity of these aftereffects via adapt-test stimulus manipulations that allowed the quantification of input from monocular, disparity-selective and disparity-invariant processing stages. Duration aftereffects showed extensive interocular transfer and are therefore generated by duration-selective mechanisms that are primarily binocular in nature. Nevertheless, a small but significant component of these aftereffects showed selectivity for eye of input. We then explored the extent to which the binocular component is mediated by neurons able to extract duration information from retinal disparity-defined stimuli which were invisible to monocular mechanisms. Disparity information alone was sufficient to generate robust duration aftereffects, indicating a duration encoding stage that is distinct from the most basic forms of binocularity. Finally, we found partial transfer across changes in the disparity which defined adapting and test durations, indicative of a third processing stage which pools duration information across disparity-selective inputs.

Bidirectional, repulsion-type duration aftereffects are consistent with the ‘channel-based’ duration encoding predicted by the response properties of duration-tuned neurons (DTNs). DTNs have been documented at multiple neural sites within a range of species^51^. Specifically, duration aftereffect magnitude varies with the similarity between adapting and test duration^30^, in keeping with model predictions rooted in activity within bandwidth-limited DTNs that varies around the neuron’s ‘preferred’ duration^30,51,52^. Although auditory DTNs have been reported in both cortical and subcortical structures^53^ reports of DTNs within the visual system have - thus far - implicated cortical sites alone^54,55^. The small but significant degree of eye input selectivity reported here (Fig. 2) is not consistent with selectivity for duration in extrastriate areas^55^ where virtually all neurons are binocular^56–58^. It is, however compatible with duration aftereffects coded via the activity of monocular neurons in V1. Although duration-tuned activity has been reported within the simple cells of area 17 of cat visual cortex^54^, the degree of binocularity exhibited by these cells remains unclear.

Alternatively, monocularity could be explained by a pre-cortical contribution to the mechanism generating the duration aftereffects themselves or that mechanism’s upstream temporal inputs. Duration-dependent firing patterns have been documented within retinal ganglion cells (RGCs)^59^ and cells within the lateral genicular nucleus (LGN)^60^. Both cell types show inhibition during stimulus presentation followed by rebound spiking response at stimulus offset. The strength of this offset response increases monotonically with increases in stimulus duration across the millisecond range. However, unlike their cortical analogue^54,61^, this duration-dependency is ‘long-pass’ rather than the bandpass type of tuning needed to produce duration aftereffects tuned around the adapting duration^30^. Thus, any subcortical contribution to duration adaptation mechanisms is more likely to occur via adaptation-induced distortion of afferent temporal information prior to its arrival at cortical bandpass DTNs.

In addition to the duration-dependent RGC and LGN cell characteristics described above, several alternative candidate mechanisms could also distort the signal within monocular channels. As discussed earlier (see Introduction), adaptation to drifting or flickering gratings induces compressive duration distortions that display multiple characteristics consistent with a neural locus within magnocellular layers of the LGN^7,8,62^. Equally, a monocular contribution could arise via duration encoding mechanisms which monitor the spatiotemporal evolution of neural activity within ‘state dependent networks’ (SDN)^63,64^ or duration-dependent changes in response magnitude within any neuron directly activated by the stimulus (so called ‘Energy Readout’ models^65–67^). Both SDN and Energy Readout are examples of ‘distributed’ duration processing that could – theoretically, at least - operate at any neural scale of stimulus-driven activity and therefore provide distorted monocular input to downstream duration aftereffect generating mechanisms.

The extensive degree of interocular transfer (Fig. 2) requires the input of binocular neurons in striate or extrastriate cortices. The fact that duration aftereffects can be generated by durations defined solely by retinal disparity (Figs 3–5) suggests that adaptation to such stimuli is generated via at least two possible routes. The first would be via a mechanism that is selective for both duration *and* retinal disparity. Figure 3’s data shows that precisely this mechanism operates during adaptation: when adapting and test durations are defined by retinal disparities that stimulate the same populations of disparity selective neurons, aftereffect strength is maximal. When adapting and test disparities stimulate distinct neural populations, duration aftereffect magnitude is markedly reduced (Figs 4 and 5). This implicates duration selectivity within disparity-selective striate or extrastriate cortices, with the later arguably more likely given the increasing prevalence of disparity selectivity (and absence of monocular neurons) in extrastriate areas^42–44,68^. Although the DTNs reported in area 17 and 18 of cat cortex could also be tuned for retinal disparity, this association has yet to be tested directly^54^.

The second route would be via a duration-selective mechanism downstream from the initial coding of retinal disparity. For example, higher-level DTNs could inherit temporal information via their disparity-selective inputs. These neurons could therefore show selectivity for duration without having any intrinsic selectivity for disparity. Figures 4 and 5’s data show that duration aftereffects are reduced but not abolished by adapt-test changes in retinal disparity, revealing significant contributions to duration encoding from both disparity-selective *and* disparity-invariant mechanisms. The latter’s contribution to duration aftereffects could, in fact be a signature of a mechanism that pools across *all* visual stimulus features. However, such an extreme form of stimulus invariance is not compatible with the aftereffect’s size-dependent spatial specificity^14^.

More plausibly, disparity invariance may be a signature of duration encoding that is selective for stimulus duration and higher-order stimulus features that are re-constructed after pooling across disparity-selective inputs. For example, human fMRI evidence shows neural selectivity within lateral occipital complex (an object selective mid-level visual area bordering V5) for depth-defined object shape but not the depth plane occupied by the object^49^. Likewise, primate V4 neurons show selectivity for 3D slant (i.e. linear disparity gradient) across changes in the shape’s distance from fixation^45^. More abstract still are objects defined by 3D curvature in depth^50^. In inferotemporal and intraparietal cortex, neurons maintain selectivity for depth-defined curvature and orientation across changes in retinal disparity and the cues defining 3D shape^46,47,69^. Could these same disparity-invariant neurons also encode stimulus duration and therefore mediate the duration aftereffect’s partial transfer across disparity? Although the answer remains unclear, duration-dependent firing patterns have been observed in monkey intraparietal cortex^70^ and recent human fMRI adaptation experiments report duration-tuned modulation of neural activity in the same region^55^.

Human psychophysical evidence also supports the concept of processing stages which pool across retinal disparity. Specifically, higher-level examples of shape, curvature, numerosity and motion perception are insensitive to changes in disparity-defined depth^71–73^. The binding of duration to stimulus features of this type would allow perceived duration to retain object specificity across changes in a range of horizontal positions^14^, depth planes and viewing angles.

In summary, we have demonstrated that duration encoding receives input from three different processing mechanisms at distinct strata within the visual processing hierarchy. An important question is the extent to which these processing stages operate in serial or parallel. The latter risks the prospect of multiple, possibly independent, duration estimates co-existing within the nervous system. This could be problematic unless they are integrated by a central mechanism which then outputs a single duration estimate. That degree of centralisation is difficult to reconcile with the stimulus specificity demonstrated in the current study and elsewhere. A perhaps more credible scenario would be for duration processing to form a serial ‘cascade’ where downstream duration encoding mechanisms apply cumulative adaptation to effects inherited from their upstream counterparts. Cascading adaptation through retinal to extrastriate sites has been reported for encoding of contrast and motion^38,74,75^. Specifically, the same spatial adaptation changes response gain in primate LGN neurons and stimulus selectivity in V1^38^, whilst motion adaptation changes responsivity in extrastriate area MT with a spatial specificity predicted by adaptation inherited from V1^74^. Although our knowledge of hierarchical interaction in the temporal domain remains primitive^76^, a picture is beginning to emerge of temporal perception underpinned by neural mechanisms whose primary function has hitherto appeared to be ostensibly spatial. Ongoing experiments will address the degree to which temporal perception can be explained by the characteristics of spatial perception, and vice versa.

## Methods

### Observers

Ten observers (seven naïve) took part in the IOT task, seven observers (five naive) in the disparity tasks. All observers gave their informed, written consent to participate, and had normal or corrected to normal vision, stereoacuity and hearing at the time of the experiment.

All experiments were conducted in accordance with the relevant guidelines and regulations, the informed written consent of each observer and received prior approval from the Research Ethics Committee at the School of Optometry and Vision Sciences, University of Bradford.

### Stimuli and Apparatus

Visual stimuli were presented on two gamma-corrected Eizo FG2421 LCD monitors with a refresh rate of 120 Hz and a resolution of 1920 × 1080. For the IOT task these were connected to a 2 × 2.8 GHz Quad-core Apple Mac Pro desktop computer running Mac OS 10.5.8, and for the disparity task they were connected to a 3 GHz E5-1660v3 8-Core HP Z440 desktop computer running Windows 8.1 Pro. All stimuli were generated using Matlab (version 7.7.0.471 or 8.4.0, Mathworks, USA) running the Psychtoolbox Extension^77^ version 3.0.8, (www.psychtoolbox.org).

The physical durations and simultaneity of all auditory and visual stimuli were verified using a dual-channel oscilloscope. Visual stimuli were viewed dichoptically through a two mirror stereoscope which allowed the left and right monitors to be monocularly viewed by left and right eyes respectively. A forehead and chin rest were used to maintain a viewing distance of 2.3 meters (one pixel subtend 0.4 arc minutes), and a head position allowing right and left eyes to remain centred in front of the stereoscope’s right and left mirrors. Prior to each experimental session, observers viewed monocular nonious lines and adjusted the tip and tilt of each mirror until these were horizontally and vertically aligned. This procedure neutralised any individual oculomotor anomalies and thus aided stable fusion through the experiment.

The auditory reference stimulus was a 500 Hz tone presented through Sennheiser HD 280 headphones. The specific visual stimuli pertaining to each task are detailed below.

### Inter-ocular transfer (IOT) task

Visual stimuli were isotropic, luminance-defined Gaussian blobs (mean luminance 74 cd/m^2^) presented at fixation against a uniform grey background of 37 cd/m^2^, whose luminance (*L*) profile was defined as follows:

$$L={L}_{mean}(1+{e}^{-(\frac{{x}^{2}}{2{{\sigma }_{stim}}^{2}})})$$

where *L*_*mean*_ is the mean luminance of the Gaussian and σ_Stim_ is the standard deviation. Throughout the IOT experiment σ_Stim_ (the size of the stimulus), was set to 1°. Fixation was maintained on a white 0.07° circular fixation marker presented at the centre of left and right screens.

### Disparity task

Visual stimuli were constructed from dynamic luminance noise stereograms viewed through the dual-mirror stereoscope. They consisted of two dichoptically presented left and right eye circular regions in which each pixel was randomly assigned a luminance value (either black or white), which was randomly updated every five frames (24 Hz) to create dynamic luminance noise. These images were identical except for a disk-shaped sub-region referred to as the ‘target zone’ (e.g. Fig. 3A). This sub-region had a diameter of 500 pixels (subtending 3.33° of visual angle when viewed at 2.3 m) within which one eye’s noise patch could be shifted laterally relative to the opposite eye’s corresponding region. The introduction/removal of this shift allowed the presentation of crossed (+ve) or uncrossed (−ve) disparity within the target zone. The surrounding dynamic noise annulus was always presented with zero disparity, as was the entire display during interstimulus intervals. To ensure that there were no monocular artefacts at either the onset of offset of the disparity-defined target, we (i) maintained constant density by ‘wrapping’ noise pixels shifted beyond the target region to the opposite side; (ii) avoided motion cues by synching changes in disparity to the noise regeneration cycle and (iii) monitored for dropped frames during stimulus presentation. The surround was bordered by a static checkered annulus which was presented to both eyes, and thus aided stable binocular fusion. Beyond this border, the luminance of the background was set to mid-grey, which was equal to the average luminance of the dynamic noise. Observers maintained fixation at the centre of the screen throughout.

### Procedure

For all experiments, a block of trials began with an initial adaptation phase consisting of 100 serially presented visual stimuli. Within a block, the duration of these stimuli was fixed at either 166 ms or 666 ms. Interstimulus interval (ISI) was randomly jittered between 500–1000 ms. The adaptation phase was a followed by a further four ‘top up’ adapting stimuli and a subsequent test phase (e.g. Fig. 1A) consisting of a fixed (333 ms) duration auditory reference stimulus and a variable duration visual test stimulus. Observers then made a two alternative forced choice (2AFC) duration discrimination judgment as to “which was longer, the visual test or auditory reference stimulus?” Visual test stimuli varied logarithmical in seven steps which were appropriately spaced relative to individual observer’s duration discrimination threshold. The order of test stimuli presentation was randomly interleaved within a method of constant stimuli. Observers responded via key press which triggered the next top-up and test cycle, until all test durations had been presented five times per block of trials.

In the IOT task, adapting and test stimuli were presented monocularly. In the “same” condition, the adapting stimulus was presented to the same eye as the visual test stimulus, and in the “different” condition (e.g. Fig. 1A) adapting and test stimuli were presented to opposite eyes. The choice of test eye was randomly assigned to each observer at the start of the experiment so that half of the observers viewed the test stimuli with their right eye and the other half their left eye. Half of the observers also performed additional conditions to ensure that the choice of test eye did not affect the magnitude of the duration aftereffect. These five observers performed all four permutations of adapt/test eye. A paired samples t-test revealed that there was no significant difference in the size of the aftereffect generated in either of the “same” test eye conditions (i.e. adapt and test right eye versus adapt and test left eye, p = 0.13), or the “different” conditions (i.e. adapt right, test left versus adapt left, test right, p = 0.54) For the remaining analysis, data was combined across equivalent ‘same’ and ‘different’ conditions.

In the retinal disparity experiments the procedure was identical to that described above with the following exceptions: all stimuli were presented binocularly and the ‘same’ vs ‘different’ manipulations pertained to situations where observers adapted and tested with stimuli defined by the same retinal disparity (adapt and test +6′ or adapt and test −24′ (Fig. 3A,C, respectively)) vs different retinal disparity (adapt +24′, test −24′ (Fig. 4A)).

Each observer completed multiple blocks for each of the two adapting durations, and for each of the ‘same’ vs ‘different’ adapting conditions, giving grand totals of 380 (IOT task) 200 (adapt and test +6′ disparity) and 210 (adapt and test −24′, adapt +24′, test −24′, disparity) repetitions per test duration, per condition.

For all experiments, the proportion of ‘test longer than reference’ responses were plotted against the physical visual test durations for each adapting duration (166 ms and 666 ms), and each experimental condition. The psychometric functions were then fitted with a logistic function of the form:

$$y=\frac{100}{1+{\exp }^{-}\frac{({\rm{x}}-\mu )}{{\rm{\theta }}}}$$

where μ is the Point of Subjective Equality (PSE): the test duration that is perceptually equivalent to the 333 ms auditory reference duration, and θ is an estimate of the discrimination threshold. For each condition, PSE values were extracted and an estimate of duration aftereffect magnitude was obtained by subtracting the PSE for the 666 ms adapting condition from the PSE obtained in the 166 ms adapting condition. 95% confidence intervals on individual and group-averaged results were obtained using non-parametric bootstrapping, with re-sampling performed at the level of an individual observer and adapt/test condition. Statistical significance of key differences between conditions was assessed using two-tailed non-parametric permutation tests.

## Data Availability

The datasets generated during and/or analysed during the current study are available in the Open Science Framework repository, https://osf.io/cqvm2/?view_only=181379d4b61748d9b92b0e978f77dee0.

## References

[1] Brunnström, K. *et al*. *Qualinet white paper on definitions of quality of experience* (European Network on Quality of Experience in Multimedia System and Services (2013).

[2] Hossfeld, T. *et al*. Initial delay vs. interruptions: Between the devil and the deep blue sea. in 1–6 (IEEE), 10.1109/QoMEX.2012.6263849 (2012).

[3] Lewis PA, Miall RC (2009). The precision of temporal judgement: milliseconds, many minutes, and beyond. Philos. Trans. R. Soc. B-Biol. Sci..

[4] Morgan MJ, Giora E, Solomon JA (2008). A single ‘stopwatch’ for duration estimation, a single ‘ruler’ for size. J. Vis..

[5] Bruno A, Cicchini GM (2016). Multiple channels of visual time perception. Curr. Opin. Behav. Sci..

[6] Kanai, R. Illusory Distortion of Subjective Time Perception. In *Subjective time: The philosophy, psychology, and neuroscience of temporality* (MIT Press, 2014).

[7] Ayhan I, Bruno A, Nishida S, Johnston A (2009). The spatial tuning of adaptation-based time compression. J. Vis..

[8] Johnston A, Arnold DH, Nishida S (2006). Spatially localized distortions of event time. Curr. Biol..

[9] Li B, Yuan X, Huang X (2015). The aftereffect of perceived duration is contingent on auditory frequency but not visual orientation. Sci. Rep..

[10] Sperry, R. W., Gazzaniga, M. S. & Bogen, J. E. Interhemispheric relationships: the neocortical commissures; syndromes of hemisphere disconnection. In: Disorders of speech, perception and symbolic behavior. Handbook of clinical neurology. No. 4. North-Holland Publishing Co., Amsterdam, pp. 273–290. ISBN 9780720472042 (1969).

[11] Handy TC, Gazzaniga MS, Ivry RB (2003). Cortical and subcortical contributions to the representation of temporal information. Neuropsychologia.

[12] Marzi CA (2004). Two brains, one clock. Trends Cogn. Sci..

[13] Westheimer G (1999). Discrimination of short time intervals by the human observer. Exp. Brain Res..

[14] Fulcher, C., McGraw, P. V., Roach, N. W., Whitaker, D. & Heron, J. Object size determines the spatial spread of visual time. *Proc. R. Soc. B-Biol. Sci*. **283** (2016). 10.1098/rspb.2016.1024 PMC4971211 27466452

[15] Li, B., Yuan, X., Chen, Y., Liu, P. & Huang, X. Visual duration aftereffect is position invariant. *Front. Psychol*. **6** (2015). 10.3389/fpsyg.2015.01536 PMC4598571 26500591

[16] New, J. J. & Scholl, B. J. Subjective time dilation: Spatially local, object-based, or a global visual experience? *J. Vis*. **9** (2009). 10.1167/9.2.4 19271914

[17] Au RK, Ono F, Watanabe K (2012). Time dilation induced by object motion is based on spatiotopic but not retinotopic positions. Front. Psychol..

[18] Burr D, Tozzi A, Morrone MC (2007). Neural mechanisms for timing visual events are spatially selective in real-world coordinates. Nat. Neurosci..

[19] Johnston A, Bruno A, Ayhan I (2011). Retinotopic selectivity of adaptation-based compression of event duration: Reply to Burr, Cicchini, Arrighi, and Morrone. J. Vis..

[20] Ortega L, Guzman-Martinez E, Grabowecky M, Suzuki S (2012). Flicker adaptation of low-level cortical visual neurons contributes to temporal dilation. J. Exp. Psychol. Hum. Percept. Perform..

[21] Zhou B, Yang S, Mao L, Han S (2014). Visual feature processing in the early visual cortex affects duration perception. J. Exp. Psychol. Gen..

[22] Ahissar M, Hochstein S (2004). The reverse hierarchy theory of visual perceptual learning. Trends Cogn. Sci..

[23] Anzai A, Peng X, Van Essen DC (2007). Neurons in monkey visual area V2 encode combinations of orientations. Nat. Neurosci..

[24] Quiroga RQ, Reddy L, Kreiman G, Koch C, Fried I (2005). Invariant visual representation by single neurons in the human brain. Nature.

[25] DiCarlo JJ, Zoccolan D, Rust NC (2012). How Does the Brain Solve Visual Object Recognition?. Neuron.

[26] Cicchini GM, Morrone MC (2009). Shifts in spatial attention affect the perceived duration of events. J. Vis..

[27] Mattes S, Ulrich R (1998). Directed attention prolongs the perceived duration of a brief stimulus. Percept. Psychophys..

[28] Osugi T, Takeda Y, Murakami I (2016). Inhibition of return shortens perceived duration of a brief visual event. Vision Res..

[29] Walker JT, Scott KJ (1981). Auditory-Visual Conflicts in the Perceived Duration of Lights, Tones, and Gaps. J. Exp. Psychol. Hum. Percept. Perform..

[30] Heron J (2012). Duration channels mediate human time perception. Proc. R. Soc. B-Biol. Sci..

[31] Gilbert CD (1983). Microcircuitry of the visual cortex. Annu. Rev. Neurosci..

[32] Hubel D, Wiesel TN (1962). Receptive fields, binocular interaction and functional architecture in the cat’s visual cortex. J. Physiol..

[33] Behar I, Bevan W (1961). The perceived duration of auditory and visual intervals: cross-modal comparison and interaction. Am. J. Psychol..

[34] Goldstone S, Lhamon WT (1974). Studies of auditory-visual differences in human time judgment. 1. Sounds are judged longer than lights. Percept. Mot. Skills.

[35] Sengpiel F, Blakemore C, Harrad R (1995). Interocular suppression in the primary visual cortex: a possible neural basis of binocular rivalry. Vision Res..

[36] Xue JT, Ramoa AS, Carney T, Freeman RD (1987). Binocular interaction in the dorsal lateral geniculate nucleus of the cat. Exp. Brain Res..

[37] Hubel D, Wiesel TN (1968). Receptive fields and functional architecture of monkey striate cortex. J. Physiol..

[38] Dhruv NT, Carandini M (2014). Cascaded effects of spatial adaptation in the early visual system. Neuron.

[39] Hubel DH, Wiesel TN (1974). Uniformity of monkey striate cortex: a parallel relationship between field size, scatter, and magnification factor. J. Comp. Neurol..

[40] Gonzalez F, Krause F, Perez R, Alonso JM, Acuna C (1993). Binocular matching in monkey visual cortex: Single cell responses to correlated and uncorrelated dynamic random dot stereograms. Neuroscience.

[41] Poggio F, Motter BC, Squatrito S, Trotter Y (1985). Responses of neurons in visual cortex (V1 and V2) of the alert macaque to dynamic random-dot stereograms. Vision Res..

[42] Poggio GF, Gonzalez F, Krause F (1988). Stereoscopic mechanisms in monkey visual cortex: binocular correlation and disparity selectivity. J. Neurosci..

[43] Uka T, DeAngelis GC (2003). Contribution of middle temporal area to coarse depth discrimination: comparison of neuronal and psychophysical sensitivity. J. Neurosci..

[44] Umeda K, Tanabe S, Fujita I (2007). Representation of stereoscopic depth based on relative disparity in macaque area V4. J. Neurophysiol..

[45] Hinkle DA, Connor CE (2002). Three-dimensional orientation tuning in macaque area V4. Nat. Neurosci..

[46] Janssen P, Vogels R, Orban GA (1999). Macaque inferior temporal neurons are selective for disparity-defined three-dimensional shapes. Proc. Natl. Acad. Sci..

[47] Durand J-B (2007). Anterior regions of monkey parietal cortex process visual 3D shape. Neuron.

[48] Sakata H (1998). Neural coding of 3D features of objects for hand action in the parietal cortex of the monkey. Philos. Trans. R. Soc. B Biol. Sci..

[49] Kourtzi Z, Kanwisher N (2001). Representation of perceived object shape by the human lateral occipital complex. Science.

[50] Orban GA (2008). Higher order visual processing in macaque extrastriate cortex. Physiol. Rev..

[51] Aubie B, Sayegh R, Faure PA (2012). Duration Tuning across Vertebrates. J. Neurosci..

[52] Ivry RB (1996). The representation of temporal information in perception and motor control. Curr. Opin. Neurobiol..

[53] Sayegh R, Aubie B, Faure P (2011). Duration tuning in the auditory midbrain of echolocating and non-echolocating vertebrates. J. Comp. Physiol. A.

[54] Duysens J, Schaafsma SJ, Orban GA (1996). Cortical off response tuning for stimulus duration. Vision Res..

[55] Hayashi, M. J. *et al*. Time Adaptation Shows Duration Selectivity in the Human Parietal Cortex. *PLoS Biol* **13** (2015). 10.1371/journal.pbio.1002262 PMC4574920 26378440

[56] Burkhalter A, Vanessen DC (1986). Processing of Color, Form and Disparity Information in Visual Areas Vp and V2 of Ventral Extrastriate Cortex in the Macaque Monkey. J. Neurosci..

[57] Hubel DH, Livingstone MS (1987). Segregation of form, color, and stereopsis in primate area 18. J. Neurosci..

[58] Zeki SM (1978). Uniformity and diversity of structure and function in rhesus monkey prestriate visual cortex. J. Physiol..

[59] Enroth-Cugell C, Pinto LH (1972). Pure central responses from off‐centre cells and pure surround responses from on‐centre cells. J. Physiol..

[60] Brooks B, Huber C (1972). Evidence for the role of the transient neural “off-response” in perception of light decrement: a psychophysical test derived from neuronal data in the cat. Vision Res..

[61] Duysens J, Orban G, Cremieux J, Maes H (1985). Velocity selectivity in the cat visual system. III. Contribution of temporal factors. J. Neurophysiol..

[62] Bruno A, Ayhan I, Johnston A (2010). Retinotopic adaptation-based visual duration compression. J. Vis..

[63] Buonomano DV, Merzenich MM (1995). Temporal Information Transformed into a Spatial Code by a Neural-Network with Realistic Properties. Science.

[64] Karmarkar UR, Buonomano DV (2007). Timing in the absence of clocks: encoding time in neural network states. Neuron.

[65] Eagleman DM, Pariyadath V (2009). Is subjective duration a signature of coding efficiency?. Philos Trans R Soc Lond B Biol Sci.

[66] Ivry RB, Schlerf JE (2008). Dedicated and intrinsic models of time perception. Trends Cogn Sci.

[67] Pariyadath V, Eagleman D (2007). The effect of predictability on subjective duration. PLoS One.

[68] DeAngelis GC, Newsome WT (1999). Organization of disparity-selective neurons in macaque area MT. J. Neurosci..

[69] Sakata H, Tsutsui K-I, Taira M (2005). Toward an understanding of the neural processing for 3D shape perception. Neuropsychologia.

[70] Leon MI, Shadlen MN (2003). Representation of time by neurons in the posterior parietal cortex of the macaque. Neuron.

[71] Bell J, Manson A, Edwards M, Meso AI (2015). Numerosity and density judgments: Biases for area but not for volume. J. Vis..

[72] Bülthoff I, Bülthoff H, Sinha P (1998). Top-down influences on stereoscopic depth-perception. Nat. Neurosci..

[73] Gheorghiu E, Kingdom FAA, Thai MT, Sampasivam L (2009). Binocular properties of curvature-encoding mechanisms revealed through two shape after-effects. Vision Res..

[74] Kohn A, Movshon JA (2003). Neuronal adaptation to visual motion in area MT of the macaque. Neuron.

[75] Larsson J, Harrison SJ (2015). Spatial specificity and inheritance of adaptation in human visual cortex. J. Neurophysiol..

[76] Heron, J., Hotchkiss, J., Aaen-Stockdale, C., Roach, N. W. & Whitaker, D. A neural hierarchy for illusions of time: duration adaptation precedes multisensory integration. *J Vis* **13** (2013). 10.1167/13.14.4 PMC3852255 24306853

[77] Brainard DH (1997). The psychophysics toolbox. Spat. Vis..

